# Coaxial nickel cobalt selenide/nitrogen-doped carbon nanotube array as a three-dimensional self-supported electrode for electrochemical energy storage†

**DOI:** 10.1039/d3ra08635f

**Published:** 2024-03-05

**Authors:** Chen Zhang, Shang Wang, Junwu Xiao

**Affiliations:** a College of Petroleum Equipment and Electrical Engineering, Dongying Vocational Institute Dongying P. R. China; b Key Laboratory of Material Chemistry for Energy Conversion and Storage, Ministry of Education, Hubei Key Laboratory of Material Chemistry and Service Failure, Department of Chemistry and Chemical Engineering, Huazhong University of Science and Technology Wuhan 430074 China dychenzhang@gmail.com

## Abstract

Herein, we propose a one-step urea pyrolysis method for preparing a nitrogen-doped carbon nanotube array grown on carbon fiber paper, which is demonstrated as a three-dimensional scaffold for constructing a nickel cobalt selenide-based coaxial array structure. Thanks to the large surface area, interconnected porous structure, high mass loading, as well as fast electron/ion transport pathway of the coaxial array structure, the nickel cobalt selenide/nitrogen-doped carbon nanotube electrode exhibits over 7 times higher areal capacity than that directly grown on carbon fiber paper, and better rate capability. The cell assembled by a nickel cobalt selenide/nitrogen-doped carbon nanotube positive electrode and an iron oxyhydroxide/nitrogen-doped carbon nanotube negative electrode delivers a volumetric capacity of up to 22.5 C cm^−3^ (6.2 mA h cm^−3^) at 4 mA cm^−2^ and retains around 86% of the initial capacity even after 10 000 cycles at 10 mA cm^−2^. A volumetric energy density of up to 4.9 mW h cm^−3^ and a maximum power density of 208.1 mW cm^−3^ are achieved, and is comparable to, if not better than, those of similar energy storage devices reported previously.

## Abstract 2

An energy storage device with a voltage window of 1.6 V is developed using three-dimensional coaxial array electrodes, and exports a volumetric energy density of 4.9 mW h cm^−3^ at 4 mA cm^−2^.

## Graphical abstract

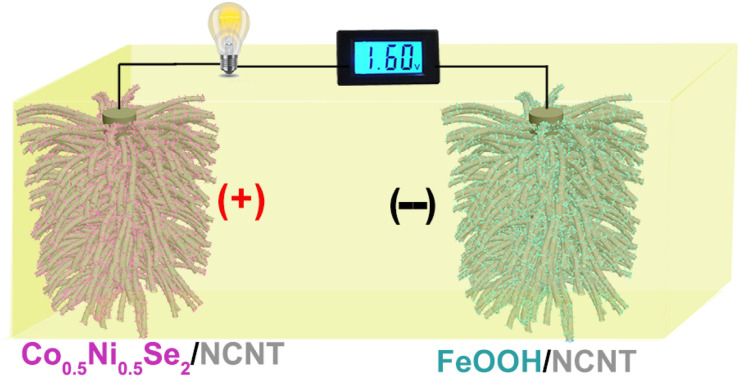

## Introductions

To meet the challenges facing battery powered vehicles, it's urgently desirable to develop energy storage devices with high specific power output and energy capacity. In spite of high efficiency and energy density, lithium ion batteries have poor cycle life and low specific power, and are unsafe due to the flammability of organic electrolytes and high reactivity of lithium electrodes.^1^ Electrochemical capacitors, also called supercapacitors, have high specific power capability and long cycle-life, but low energy density limits the wide applications.^2,3^ Irrespective of batteries and electrochemical capacitors, their electrochemical performances are much below those required by future electric vehicles. This drives revolutionary advances in the design and fabrication of a hybrid electrochemical energy storage system with battery-type and capacitive charge storage. This hybrid electrochemical energy storage device, also called supercapattery,^4,5^ can combine the voltage window of the capacitive and battery electrodes, allowing for delivering high energy density but maintaining the high power output and long cycle life of a capacitive electrode. The energy storage performance of a supercapattery is to some degree dependent on the electrochemical performance and voltage window of the electrode materials, especially battery-type materials which mainly contribute to the capacity *via* their rich redox reactions. Hence, it's of critical significance to pursue battery-type electrode materials with high capacity, wide voltage window, and low equivalent series resistance.

By virtue of their dynamic faradaic redox reaction and low cost, transition metal (Fe, Co, Ni, Mo, V, *etc.*) compounds have been demonstrated as a class of prospective battery-type electrode materials.^6^ Binary transition metal oxides and sulfides, in particular, have demonstrated higher electric conductivity and reversible capacity as compared to monometallic compounds, and thus garner considerable attention.^7–9^ Selenium is in the same group as oxygen and sulfur, and the metallic character of transition metal selenides, which contrast sharply with the semiconducting nature of the oxide and sulfide, makes it a potentially promising material as an advanced electrode.^10–12^ Selenides, such as, cobalt selenides, nickel selenides, nickel cobalt selenides, *etc.*, have previously been shown to perform admirably in terms of energy conversion and storage,^13–25^ especially for multi-metallic species.

Aside from active components, electrode structure is a crucial factor in determining the electrochemical performance. Three-dimensional electrodes with hierarchical porous structure, high active material loading, large surface area, as well as tunable free volume show much better electrochemical performance than common slurry-cast electrodes.^26,27^ To date, the large obstacle is to design an ideal electroconductive scaffold including porous metals,^28–30^ metal oxides/nitrides/sulfides,^31–33^ carbon matrices,^34,35^*etc.* Carbon materials (nanotubes, nanofibers, and nanofoams) are thought as a promising candidate for three dimensional scaffolds due to low cost, lightweight, and good chemical stability. However, to date, the primary manufacture of graphitized carbon matrices uses explosive and combustible gas (methane, ethylene, and hydrogen) by chemical vapor deposition (CVD), which greatly limits large-scale application. Moreover, a direct thermal decomposition of carbon precursors results in low degree of crystallinity and graphitization. Hence, it remains a great challenge to fabricate a promising three-dimensional electrode that resembles highly electrochemical active components and a highly conducting, stable scaffold.

We herein reported a facile urea pyrolysis method to synthesize highly graphitized nitrogen-doped carbon nanotube (NCNT) array grown on carbon fiber paper (CFP) as a three-dimensional scaffold. Nickel cobalt selenide in the formula of Co_0.5_Ni_0.5_Se_2_ and iron oxyhydroxide (FeOOH) active components were deposited at the NCNT to form the coaxial array structure, namely Co_0.5_Ni_0.5_Se_2_/NCNT and FeOOH/NCNT, respectively. These coaxial three-dimensional electrodes are assembled into energy storage device with a wide voltage window of 1.6 V and high volumetric capacity of 22.5 C cm^−3^ at 4.0 mA cm^−2^, resulting in a maximum energy density reaching 4.9 mW h cm^−3^.

## Experimental

### Construction of nitrogen-doped carbon nanotube (NCNT) array

The synthetic method is described as follows: In a typical process, 0.15 M of FeCl_3_·6H_2_O and 1.0 M of NaNO_3_ were first dissolved in 40 mL of deionized water. The pH value was adjusted to 1.5 using hydrochloric acid (37 wt%). Carbon fiber paper (CFP) was vertically hanged in above solution and was transferred into a Teflon-lined stainless-steel autoclave. After being reacted at 95 °C for 12 h, the samples were taken out and washed by deionized water. Followed by the pyrolysis at 1000 °C under Ar atmosphere for 1 h, nitrogen-doped carbon nanotube (NCNT) array are nucleated and grown at carbon fiber paper, when 10 g of urea and the samples located at the front end and center of the tube furnace. Finally, the iron catalysts were removed in HCl solution (2.0 M) at 60 °C for 6 h to form NCNT array.

### Synthesis of Co_0.5_Ni_0.5_(OH)_2_/NCNT, Co_0.5_Ni_0.5_Se_2_/NCNT, and FeOOH/NCNT

Coaxial Co_0.5_Ni_0.5_(OH)_2_/NCNT electrode with an optimal Co/Ni molar ratio of 1 : 1 was synthesized using a mixture of CoCl_2_ (50 mM) and NiCl_2_ (50 mM) solution through a potentiostatic deposition in a three-electrode, single-compartment electrochemical cell.^9,36^ NCNT array scaffold, Pt mesh, and Ag/AgCl(Saturated KCl) were the working, counter, and reference electrodes, respectively. Co_0.5_Ni_0.5_(OH)_2_ nanosheets are deposited at the NCNT surface to form coaxial Co_0.5_Ni_0.5_(OH)_2_/NCNT after being executed at −0.8 V *vs.* Ag/AgCl for 30 min.

Co_0.5_Ni_0.5_Se_2_/NCNT was prepared through a selenization of Co_0.5_Ni_0.5_(OH)_2_/NCNT. The details were described below: 0.1 g of selenium and 3.0 g of sodium hydroxide were dissolved into 25 mL of deionized water at 85 °C, and then transferred into a Teflon-lined stainless-steel autoclave. Co_0.5_Ni_0.5_(OH)_2_/NCNT was subsequently immersed into above solution and kept at 180 °C for 12 h. After cooling down to room temperature, the samples, namely Co_0.5_Ni_0.5_Se_2_/NCNT, were washed by ethanol and dried up.

Coaxial FeOOH/NCNT structure was synthesized by using an anodic deposition method in a two-electrode, single-compartment electrochemical cell.^9^ NCNT array scaffold and Pt mesh were the working and counter electrodes, respectively. The electrolyte is 20 mM of FeCl_2_ aqueous solution. After 20 minutes at 1.5 V and 75 °C, FeOOH active components were deposited on the NCNT, resulting in forming coaxial FeOOH/NCNT.

### Materials characterizations

The morphologies of the samples were examined by scanning electron microscopy (SEM) using JEOL JSM-6700F at an accelerating voltage of 5 kV and transmission electron microscopy (TEM) on a JEOL 2010F microscope operating at 200 kV. The crystal phases of the samples were carried out using X-ray diffraction (XRD) performed on a Philips PW-1830 X-ray diffractometer with Cu kα irradiation (*λ* = 1.5406 Å). X-ray photoelectron spectroscopy (XPS) was measured on a PerkinElmer model PHI 5600 XPS system with a resolution of 0.3–0.5 eV from a monochromated aluminum anode X-ray source with Kα radiation (1486.6 eV). The Co/Ni molar ratio in the samples was determined by X-ray fluorescence spectroscopy (XRF) carried out on EAGLE III (EDAX Inc). Brunauer–Emmett–Tell (BET) surface area of the samples were obtained from nitrogen sorption isotherms at 77 K and were carried out on Micromeritics ASAP 2460 instrument.

### Electrochemical characterizations

The electrochemical performance was evaluated on a CHI 660E electrochemical workstation using cyclic voltammetry (CV), chronopotentiometry (CP), and electrochemical impedance spectroscopy (EIS) techniques in a three-electrode configuration. Three-dimensional electrodes with an area of 1 cm^2^, Pt foil, and Hg/HgO (1.0 M KOH solution) were used as working electrode, counter electrode, and reference electrode, respectively. The electrolyte is 1.0 M KOH solution. The cell was assembled by using Co_0.5_Ni_0.5_Se_2_/NCNT positive electrode, FeOOH/NCNT negative electrode, and KOH (2 M)/polyvinyl alcohol (PVA) gel electrolyte. The overall thickness (*h*) of the device was 0.08 cm. The specific capacity from galvanostatic charge–discharge curves is obtained according to the eqn (1) and (2):1
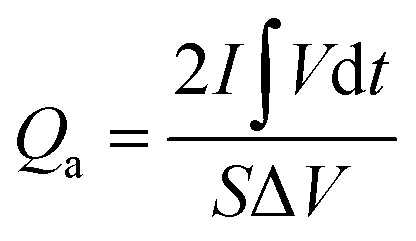
2
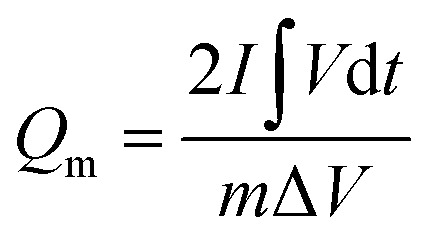
where *Q*_a_ (C cm^−2^) and *Q*_m_ (C g^−1^) represent the specific capacity depending on the electrode area and active component mass, respectively. *I*, *S*, *t*, Δ*V*, and *m* are the discharge current (A), electrode area (cm^2^), discharge time (s), voltage window (V), active component mass (g), respectively.

The specific capacity from CV curves is calculated according to the eqn (3):3
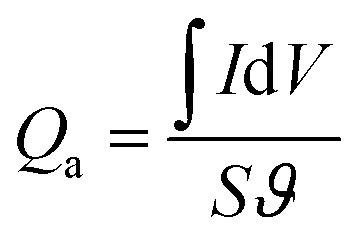
where *ϑ* is scan rate (mV s^−1^). The volumetric energy density (*E*, mW h cm^−3^), power density (*P*, mW cm^−3^) are obtained according to the eqn (4) and (5):^37–39^4
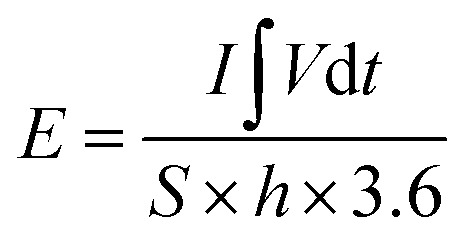
5
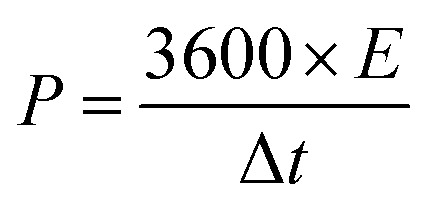
where *h* and Δ*t* are the thickness (0.08 cm) of the device and discharge time (s), respectively.

## Results and discussion

**Fig. 1:**
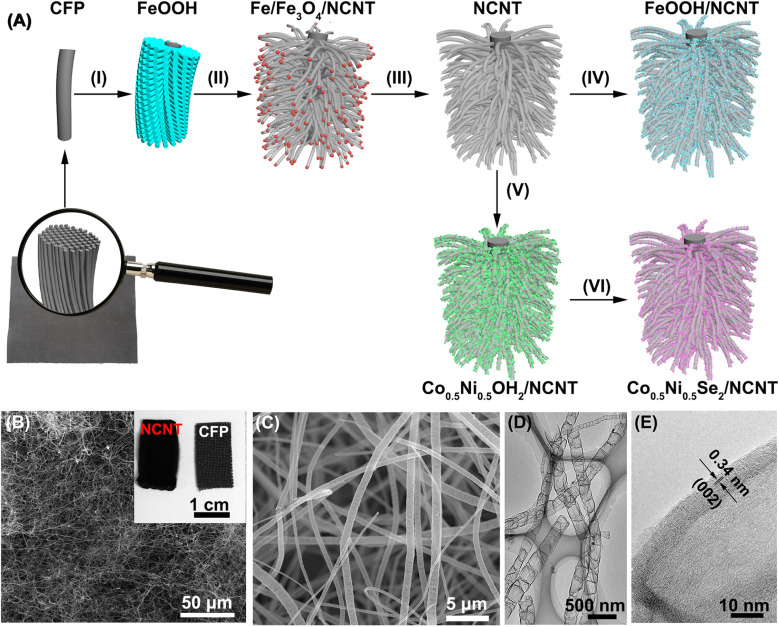
(A) Schematic diagram showing the fabrication process of three dimensional coaxial array electrodes. (B) SEM image of Fe/Fe_3_O_4_/NCNT (inset: digital images of CFP and Fe/Fe_3_O_4_/NCNT). (C) SEM, (D) TEM, and (E) HRTEM images of NCNT array. (CFP: carbon fiber paper; NCNT: nitrogen-doped carbon nanotube).

Fig. 1A depicts the formation process of coaxial three-dimensional electrodes. First of all, well-aligned FeOOH short nanorods were uniformly grown on CFP *via* the hydrolysis of FeCl_3_ (Step I in Fig. 1A and S1†).^40^ FeOOH is converted into iron nanocrystals during urea pyrolysis process, and as catalytic sites further initiate the nucleation and growth of NCNT using the pyrolysis products *via* a chemical vapor deposition (Step II in Fig. 1A).^41^ It is evidenced by diffraction peaks of iron nanocrystals at 2*θ* = 43.7° and 63.4° appearing in the XRD pattern (Fig. S2†) and the majority of iron nanoparticles locating at the tip of NCNT (Fig. S3A and B†). The XRD pattern also shows two diffraction peaks at 2*θ* = 30.1° and 35.4°, which assign to Fe_3_O_4_. It is probably because a few iron nanocrystals are likely to oxidize without the protection of carbon layer when exposed to air.^42^ Photograph and SEM images in Fig. 1B and S3† reveal the dense growth of NCNT array on CFP, and its length depends on the pyrolysis times and can reach several hundred microns after 60 min reaction. During the subsequent acid leaching process, iron species were completely dissolved (Step III in Fig. 1A), as illustrated by the disappearance of the Fe and Fe_3_O_4_ phase in the XRD pattern (Fig. S2†). While the bamboo-like structure of NCNT array is well conserved, as displayed by Fig. 1C and D. High-resolution TEM image in Fig. 1E reveals the well-defined lattice fringes at a spacing of 0.34 nm that match to the (002) plane of graphite, indicative of high crystallization and graphitization degrees of NCNT array.

**Fig. 2:**
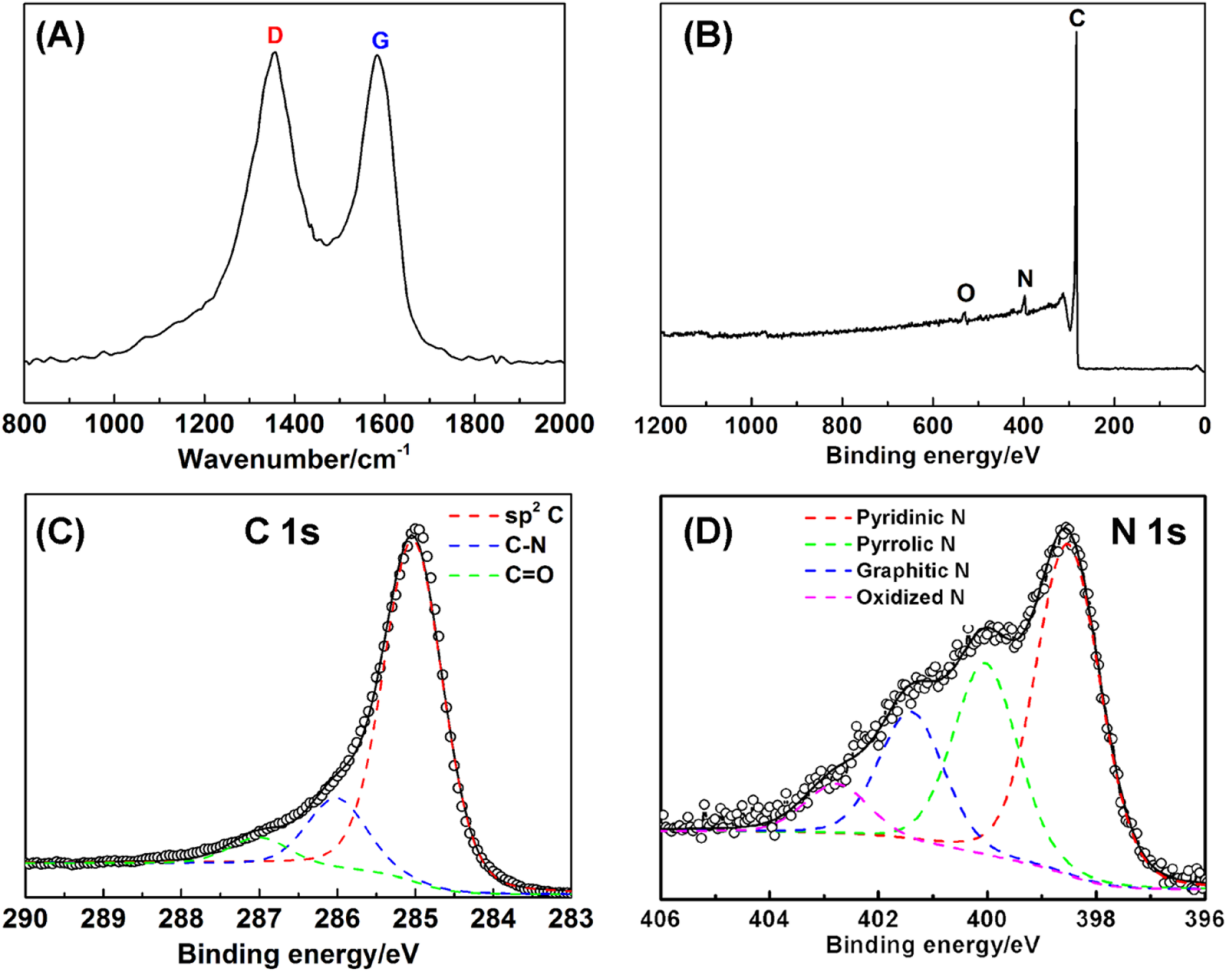
(A) Raman spectrum (B) XPS full spectrum, and (C and D) high resolution C 1s and N 1s XPS spectra of NCNT array.

Raman and XPS techniques are used to further characterize the graphitization degree and bonding configuration of NCNT. The Raman spectrum in Fig. 2A reveals the graphitic D and G bands at 1355 cm^−1^ and 1583 cm^−1^, respectively, with the intensity ratio (*I*_D_/*I*_G_) close to 1.04, most likely due to oxygen and nitrogen doping into the sp^2^ carbon structure. It is manifested by the appearance of oxygen and nitrogen signals in XPS spectra (Fig. 2B). The absence of iron signal verifies the complete dissolution of iron species during the acid etching process. The C 1s XPS signal is deconvoluted into three peaks at 285.0 eV, 285.8 eV, and 287.0 eV, which are ascribed to sp^2^C, C–N and C=O, respectively (Fig. 2C).^43^ High resolution N 1s XPS spectrum is fitted into four peaks at the binding energies of 398.1 eV, 400.2 eV, 401.4 eV, and 402.7 eV, ascribing to pyridinic, pyrrolic, graphitic, and oxidized N atoms with a percentage of 44.2%, 27.8%, 19.9%, and 8.1%, respectively (Fig. 2D). Low O/C (<1.0%) and N/C (<3.8%) ratios in the NCNT verify a high degree of graphitization degree, making it a promising three-dimensional scaffold.

**Fig. 3:**
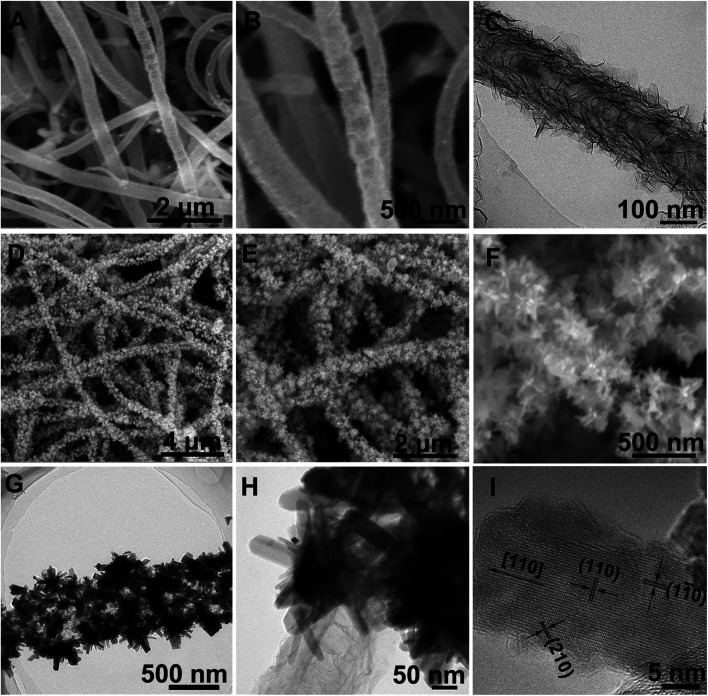
SEM and TEM images of Co_0.5_Ni_0.5_(OH)_2_/NCNT (A–C) and Co_0.5_Ni_0.5_Se_2_/NCNT (D–I).

Using NCNT array as a scaffold, electrochemically active components, such as, Co_0.5_Ni_0.5_(OH)_2_, Co_0.5_Ni_0.5_Se_2_, and FeOOH, were deposited at the surface to form the coaxial structure. As seen from Fig. 3A–C and Step IV in Fig. 1A, the thin Co_0.5_Ni_0.5_(OH)_2_ nanosheets were uniformly grown at the NCNT using a mixture of CoCl_2_ and NiCl_2_ (Co/Ni = 1 : 1) solution, resulting in forming the coaxial Co_0.5_Ni_0.5_(OH)_2_/NCNT array.^9,36^ The Co_0.5_Ni_0.5_(OH)_2_ loading in the coaxial array increases by a factor of >3 as compared to that directly deposited on carbon fiber paper, which is probably ascribed to larger surface area (38.0 m^2^ g^−1^) of NCNT array than that (9.8 m^2^ g^−1^) for carbon fiber paper (Fig. S4†). After a selenization process, Co_0.5_Ni_0.5_(OH)_2_ component in the coaxial array are converted into the selenides, which is demonstrated by the diffraction peaks agreeing well with that of CoSe_2_ (JCPDS 00-029-1417, Fig. S5†). The X-ray fluorescence (XRF) confirms the co-existence of Co and Ni species in the Co_0.5_Ni_0.5_Se_2_/NCNT with a Co/Ni and (Co + Ni)/Se ratio of 0.96 and 0.51, in well agreement with that in the reactants and chemical formula of CoSe_2_. As displayed by SEM images (Fig. 3D and E), Co_0.5_Ni_0.5_Se_2_/NCNT also displays the coaxial array structure. However, distinguished from thin Co_0.5_Ni_0.5_(OH)_2_ nanosheets in Co_0.5_Ni_0.5_(OH)_2_/NCNT, flower-like Co_0.5_Ni_0.5_Se_2_ in Co_0.5_Ni_0.5_Se_2_/NCNT is composed of short nanorods (Fig. 3F–H). High-resolution TEM image in Fig. 3I shows the short nanorods grow along the [110] direction. The Co_0.5_Ni_0.5_(OH)_2_ nanosheets and Co_0.5_Ni_0.5_Se_2_ nanorods are also explored for being grown at carbon fiber paper *via* a similar process with Co_0.5_Ni_0.5_(OH)_2_/NCNT and Co_0.5_Ni_0.5_Se_2_/NCNT (Fig. S6†). In addition, the FeOOH component with a mass loading of 3.0 mg cm^−2^ is deposited at the NCNT array scaffold to produce the coaxial array structure (Step VI in Fig. 1A, S7 and S8†), when the deposition time is 20 min.

**Fig. 4:**
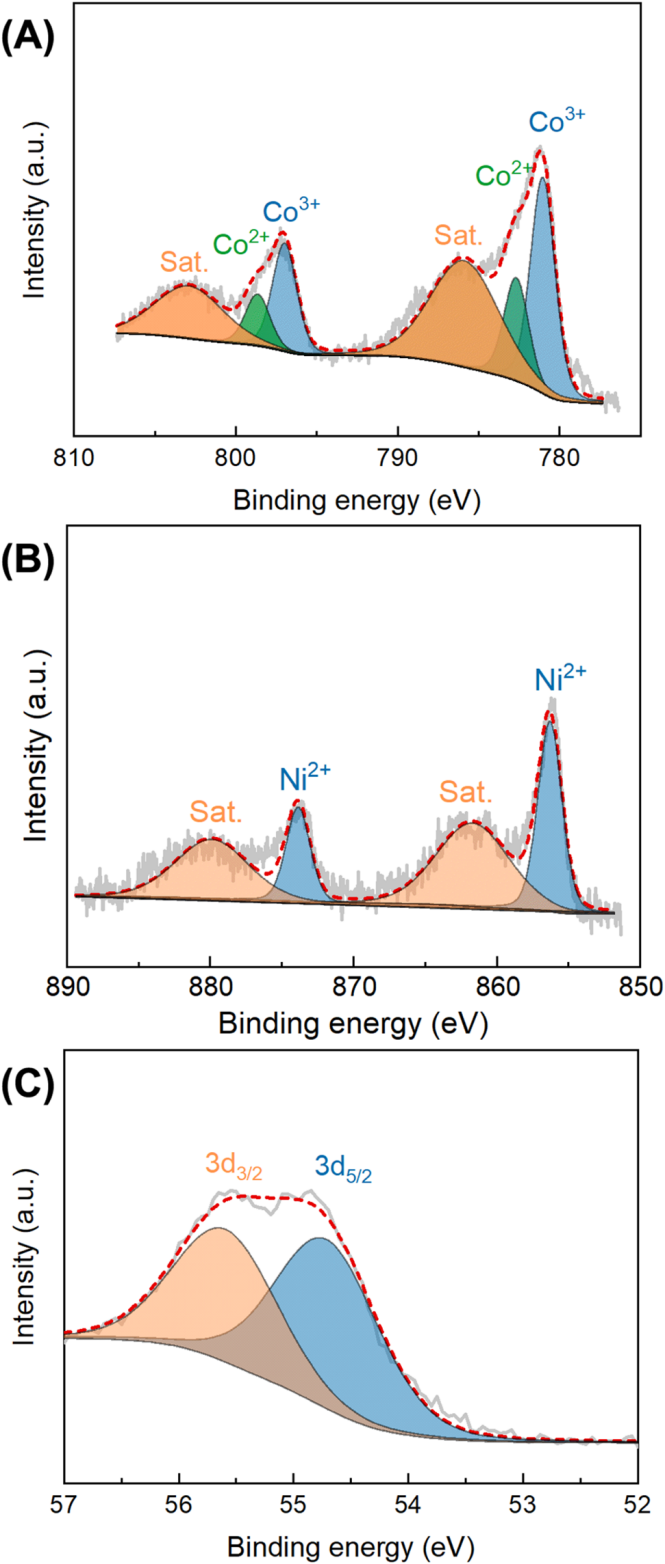
The Co 2p (A), Ni 2p (B), and Se 3d (C) fine XPS spectra for Co_0.5_Ni_0.5_Se_2_/NCNT.

The chemical composition and bonding configuration of Co_0.5_Ni_0.5_Se_2_/NCNT were investigated by using XPS technology. The XPS signals in Fig. S9† suggest the existence of Co, Ni, Se, C, N, and O species in Co_0.5_Ni_0.5_Se_2_/NCNT. High resolution Co 2p XPS spectra display four peaks at 781.0 eV, 782.7 eV, 797.0 eV, and 798.7 eV, which are attributed to the 2p_3/2_ signals for Co^3+^ and Co^2+^ and the 2p_1/2_ signals for Co^3+^ and Co^2+^, respectively.^44^ The additional two peaks located at 785.9 eV and 802.9 eV assign to the satellite peaks of Co 2p_3/2_ and Co 2p_1/2,_ respectively. The peaks at binding energies of 856.3 eV and 873.9 eV in Ni 2p XPS signals belong to Ni^2+^ 2p_3/2_ and Ni^2+^ 2p_1/2_, respectively (Fig. 4B).^45^ The corresponding satellite peaks locate at 861.7 eV and 879.9 eV. In Se 3d XPS signals, the peaks at 54.8 eV and 55.7 eV are the Se 3d spin-orbits (3d_5/2_ and Se 3d_3/2_) of metal–selenium bond (Fig. 4D).^46^ The O 1s, C 1s, and N 1s fine XPS signals of Co_0.5_Ni_0.5_Se_2_/NCNT verify that carbon, nitrogen, and oxygen species originate from NCNT and carbon fiber paper components (Fig. S10–S12†), and show the similar environments with that for NCNT array.

**Fig. 5:**
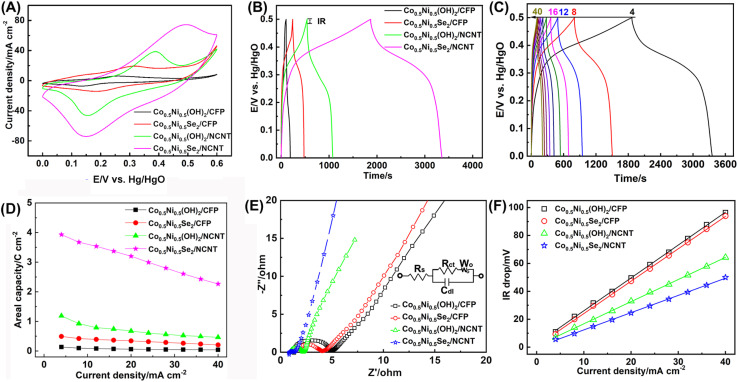
Electrochemical Performance of Co_0.5_Ni_0.5_(OH)_2_/CFP, Co_0.5_Ni_0.5_Se_2_/CFP, Co_0.5_Ni_0.5_(OH)_2_/NCNT, and Co_0.5_Ni_0.5_Se_2_/NCNT measured in a three electrode configuration. (A) CV curves at a scan rate of 20 mV s^−1^. (B) The galvanostatic charge/discharge curves at 4 mA cm^−2^. (C) The galvanostatic charge/discharge curves of Co_0.5_Ni_0.5_Se_2_/NCNT at 4–40 mA cm^−2^. (D) The rate capability performance at 4–40 mA cm^−2^. (E) EIS Nyquist plots measured at an open circuit potential in the frequency range of 1000 kHz to 0.01 Hz with a ac perturbation of 5 mV (inset: the equivalent circuit diagram proposed for analysis of the EIS data). (F) The *V*_drop_*versus* current density curves.

The electrochemical properties of Co_0.5_Ni_0.5_Se_2_/NCNT were tested in a typical three electrode configuration. The Co_0.5_Ni_0.5_(OH)_2_/CFP, Co_0.5_Ni_0.5_Se_2_/CFP, and Co_0.5_Ni_0.5_(OH)_2_/NCNT electrodes are given for comparison. As depicted by Fig. 5A, they all have a pair of redox peak corresponding to the redox reaction of Co^2+^/Co^3+^ and Ni^2+^/Ni^3+^. The nonlinear galvanostatic charge/discharge curves in Fig. 5B further suggest a faradaic process during discharging/charging, which is difference from quasi-rectangular CV curve and linear galvanostatic charge/discharge curve for NCNT array scaffold mainly contributed by electrical double layer capacitance (Fig. S13†). Note that the discharge time follows the order of Co_0.5_Ni_0.5_(OH)_2_/CFP < Co_0.5_Ni_0.5_Se_2_/CFP < Co_0.5_Ni_0.5_(OH)_2_/NCNT < Co_0.5_Ni_0.5_Se_2_/NCNT at 4 mA cm^−2^. An areal capacity at 4 mA cm^−2^ is 3.9 C cm^−2^ for Co_0.5_Ni_0.5_Se_2_/NCNT and 1.2 C cm^−2^ for Co_0.5_Ni_0.5_(OH)_2_/NCNT, which are two order of magnitude higher than NCNT array scaffold (21.0 mC cm^−2^). It reveals the capacity of the coaxial array electrodes mainly originates from battery-type charge storage of Co_0.5_Ni_0.5_(OH)_2_ and Co_0.5_Ni_0.5_Se_2_ materials rather than capacitive charge storage of NCNT array scaffold. Moreover, the coaxial array electrodes have over 7 times higher areal capacity than 0.5 C cm^−2^ for Co_0.5_Ni_0.5_Se_2_/CFP and 0.1 C cm^−2^ for Co_0.5_Ni_0.5_(OH)_2_/CFP, probably due to large surface area and good electric conductivity of NCNT array scaffold. Fig. 5C shows the charge/discharge profiles of Co_0.5_Ni_0.5_Se_2_/NCNT at 4–40 mA cm^−2^, of which the symmetric character implies good rate capability. It is consolidated by the high retention rate of the areal capacity when increasing the discharge current from 4 mA cm^−2^ to 40 mA cm^−2^ (Fig. 5D), where 57.6% of the capacity is preserved for Co_0.5_Ni_0.5_Se_2_/NCNT, in contrast to 39.0% for Co_0.5_Ni_0.5_(OH)_2_/NCNT, 41.2% for Co_0.5_Ni_0.5_Se_2_/CFP, and 29.3% for Co_0.5_Ni_0.5_(OH)_2_/CFP.

Note that a specific capacity of about 280 C g^−1^ at ∼5 A g^−1^ is achieved for Co_0.5_Ni_0.5_(OH)_2_/NCNT, much higher than 168 C g^−1^ for Co_0.5_Ni_0.5_(OH)_2_/CFP, may since NCNT array with large surface area and hierarchical structure promote the exposure of active component to the electrolyte and rapid ion/electron transport. When Co_0.5_Ni_0.5_Se_2_ component replaced of Co_0.5_Ni_0.5_(OH)_2_ in the coaxial array electrode, the specific capacity shows a notable increase and approaches to over 650 C g^−1^ and 411 C g^−1^ for Co_0.5_Ni_0.5_Se_2_/NCNT at ∼1 A g^−1^ and 5 A g^−1^, respectively (Fig. S14†), suggesting better electrochemical performance of Co_0.5_Ni_0.5_Se_2_ than Co_0.5_Ni_0.5_(OH)_2_. Moreover, the specific capacity of Co_0.5_Ni_0.5_Se_2_/NCNT stands the top level among the best-performing nickel cobalt selenide electrodes reported recently (Table S1†). The good long-term stability performance of Co_0.5_Ni_0.5_Se_2_/NCNT is demonstrated by low to 5.9% of the initial capacity loss when being repetitively charged/discharged at 20 mA cm^−2^ for 5000 cycles (Fig. S15†), in contrast to 11.6% loss for Co_0.5_Ni_0.5_Se_2_/CFP, 12.9 loss for Co_0.5_Ni_0.5_(OH)_2_/NCNT, and 39.7% for Co_0.5_Ni_0.5_(OH)_2_/CFP.

The coaxial Co_0.5_Ni_0.5_Se_2_/NCNT array electrode exhibits an impressive electrochemical performance, which is attributed to three factors listed below. First of all, large surface area and porous features of NCNT array are favorable for loading more active components, such as, 0.8 mg cm^−2^ for Co_0.5_Ni_0.5_(OH)_2_/CFP, 2.7 mg cm^−2^ for Co_0.5_Ni_0.5_(OH)_2_/NCNT, 1.8 mg cm^−2^ for Co_0.5_Ni_0.5_Se_2_/CFP, and 5.5 mg cm^−2^ for Co_0.5_Ni_0.5_Se_2_/NCNT, and thus increase the areal capacity. Secondly, it is evidenced that the charge storage has nothing to do with selenium species and instead results from rich Co^2+^/Co^3+^ and Ni^2+^/Ni^3+^ redox reactions.^11^ Therefore, the higher electrochemical activity and electric conductivity of the selenides than hydroxides lead to the improved charge storage performance. Thirdly, NCNT array scaffold promotes rapid electron/ion transport, as seen from electrochemical impedance spectroscopy (EIS) and equivalent series resistance (ESR). EIS curves in Fig. 5E reveal that the charge transfer resistance (*R*_ct_) reduce from 2.01 ohm for Co_0.5_Ni_0.5_Se_2_/CFP to 0.27 ohm for Co_0.5_Ni_0.5_Se_2_/NCNT, and 3.28 ohm for Co_0.5_Ni_0.5_(OH)_2_/CFP to 1.15 ohm for Co_0.5_Ni_0.5_(OH)_2_/NCNT. The ESR derived from the curves of *V*_drop_*versus* current density agree well with the EIS results (Fig. 5F), suggesting faster electron and ion transport characteristics of the coaxial array electrode, in comparison with Co_0.5_Ni_0.5_(OH)_2_ and Co_0.5_Ni_0.5_Se_2_ components directly grown on carbon fiber paper.

**Fig. 6:**
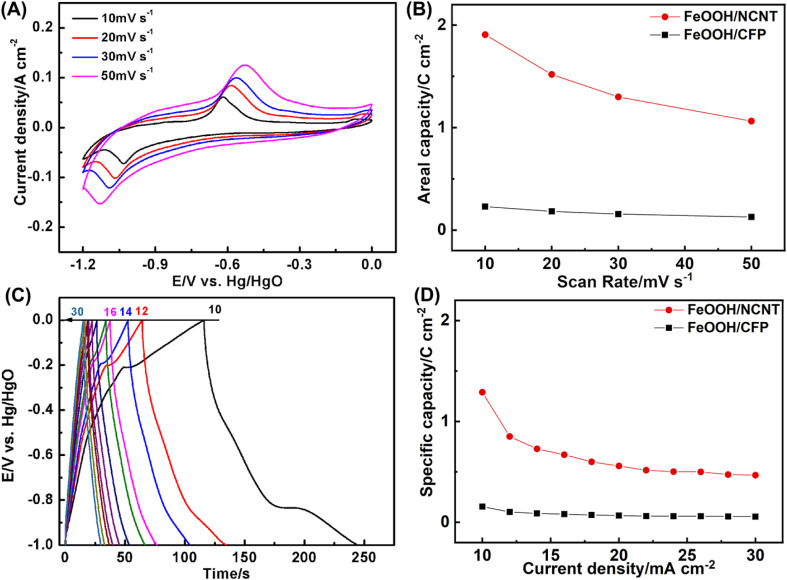
(A) CV curves of FeOOH/NCNT at the scan rates of 10, 20, 30, and 50 mV s^−1^ (B) The areal capacity of FeOOH/CFP and FeOOH/NCNT calculated according to CV curves. (C) The galvanostatic charge/discharge curves of FeOOH/NCNT at 10–30 mA cm^−2^. (D) The areal capacity of FeOOH/CFP and FeOOH/NCNT calculated according to galvanostatic charge/discharge curves.

Fig. 6 depicts the electrochemical performance of the coaxial FeOOH/NCNT array electrode performed in a three-electrode configuration. As depicted in Fig. 6A, a pair of redox peak appears in the CV curves at the potential range of −1.2–0.0 V *vs.* Hg/HgO, which belongs to the conversion of Fe^3+^/Fe^2+^. The capacity calculated according to CV curves is 1.90 C cm^−2^ at 10 mV s^−1^ and 1.06 F cm^−2^ at 50 mV s^−1^ (Fig. 6B), which increase by a factor of ∼8 as compared to the FeOOH/CFP electrode. Fig. 6C shows galvanostatic charge/discharge curves in the voltage window of 1.0 V.^47–49^ The capacity reaches 1.29 C cm^−2^ for FeOOH/NCNT at 10 mA cm^−2^. Despite a significant drop when the discharge current is increased to 30 mA cm^−2^, the capacity of FeOOH/NCNT is still substantially larger than that of FeOOH/CFP, further illustrating the improved electrochemical performance of the coaxial array electrode.

**Fig. 7:**
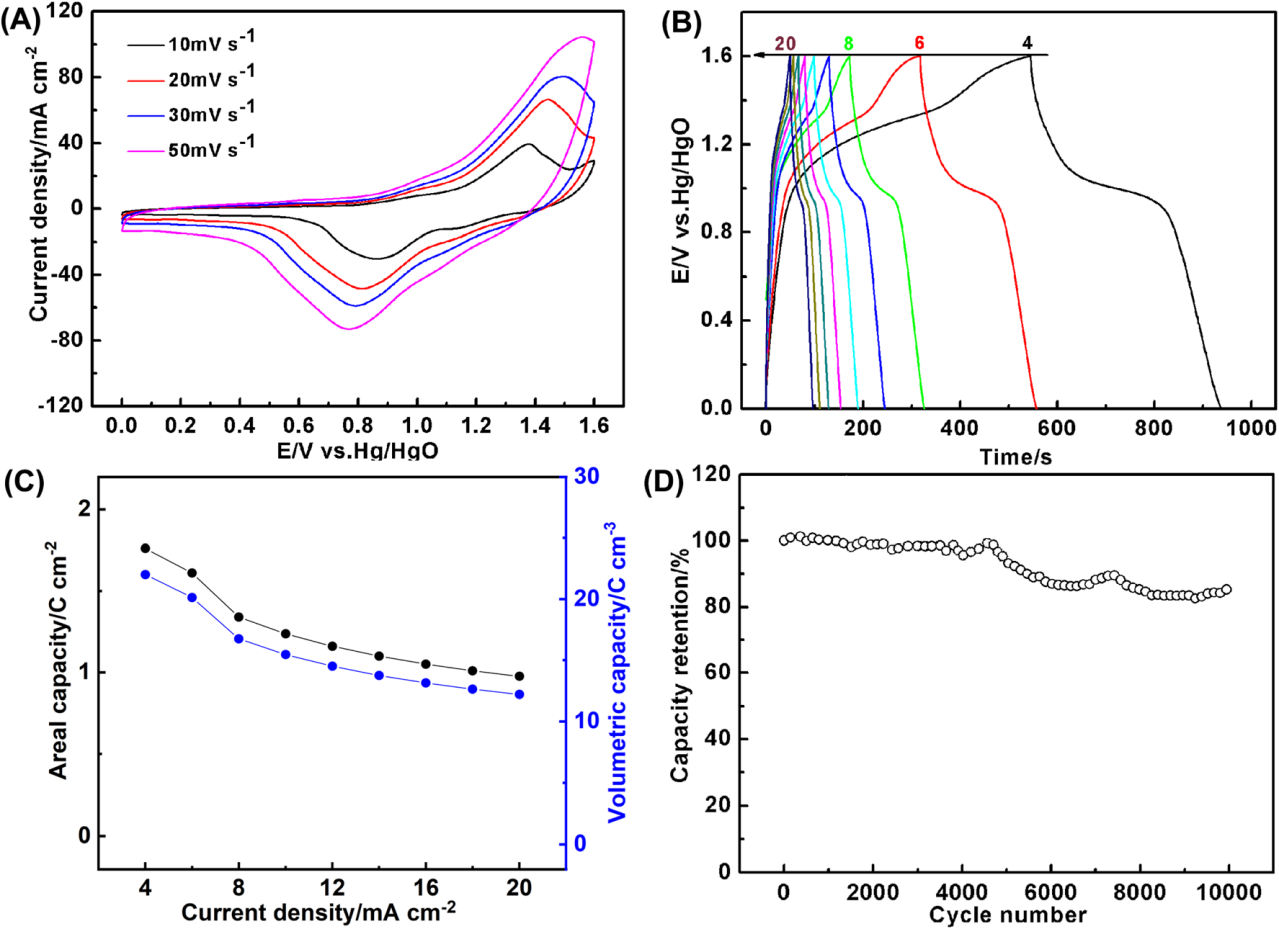
(A) CV curves (B) the galvanostatic charge/discharge curves, (C) the areal and volumetric capacity, and (D) long-term cycling stability at 10 mA cm^−2^ of Co_0.5_Ni_0.5_Se_2_/NCNT//FeOOH/NCNT cell.

In views of good electrochemical performance of the coaxial array electrodes, a cell with the thickness of 0.08 cm was assembled by using Co_0.5_Ni_0.5_Se_2_/NCNT positive electrode and FeOOH/NCNT negative electrode. CV curves in Fig. 7A reveal that Co_0.5_Ni_0.5_Se_2_/NCNT//FeOOH/NCNT cell can stably scan from 0 V to 1.6 V at a scan rate of 10–50 mV s^−1^ (Fig. 7A), as evidenced by the successful charge/discharge in the potential of 0–1.6 V (Fig. 7B), suggesting the voltage window extending to 1.6 V. Fig. 7C shows a specific capacity of Co_0.5_Ni_0.5_Se_2_/NCNT//FeOOH/NCNT cell at 4–20 mA cm^−2^. The capacity approaches to 1.8 C cm^−2^ (0.5 mA h cm^−2^ and 207.2 C g^−1^) and 22.5 C cm^−3^ at 4 mA cm^−2^, respectively. Even when the discharge current is increased to 20 mA cm^−2^, the cell still exports a capacity of 1.0 C cm^−2^ (0.3 mA h cm^−2^ and 114.8 C g^−1^) and 12.5 C cm^−3^. The capacity is comparable to, if not better than, those of previously reported nickel cobalt selenide-based energy storage devices including (Ni, Co)_0.85_Se//porous graphene (0.95 C cm^−2^ at 1 mA cm^−2^),^50^ (Ni_0.1_Co_0.9_)_9_S_8_@NF//rGO@NF (42.6 C g^−1^ at 0.2 A g^−1^),^20^ H–NiCoSe_2_//AC (168 C g^−1^ at 0.2 A g^−1^),^51^ CoNiSe_2_/CoNiSe_2_//CoNiO_2_/CoNiO_2_ (16.2 C cm^−3^ at 50.9 mA cm^−3^),^52^ (Ni, Co)Se_2_/NiCo-LDH//porous carbon (163.2 C g^−1^ at 2 A g^−1^),^53^ NiCo_2_Se_4_//BiSe (308.7 C g^−1^ at 2 A g^−1^),^54^ Ni_4.5_Co_4.5_-Se/NPCC//Fe_3_C/CF (113.7 C g^−1^ at 1 A g^−1^),^55^ NiSe_2_/CoSe_2_//N, S-rGO (257.5 C g^−1^ at 0.5 A g^−1^),^56^*etc.* The long-term durability is examined *via* the repetitive charge–discharge process at 10 mA cm^−2^. As depicted by Fig. 7D, the capacity shows no decline in the initial 5000 cycles. Merely 14.0% of the capacity is lost even after 10 000 repetitive cycles, which is probably due to the component transformation from the selenide to hydroxide and poor electric conductivity of FeOOH component.

**Fig. 8:**
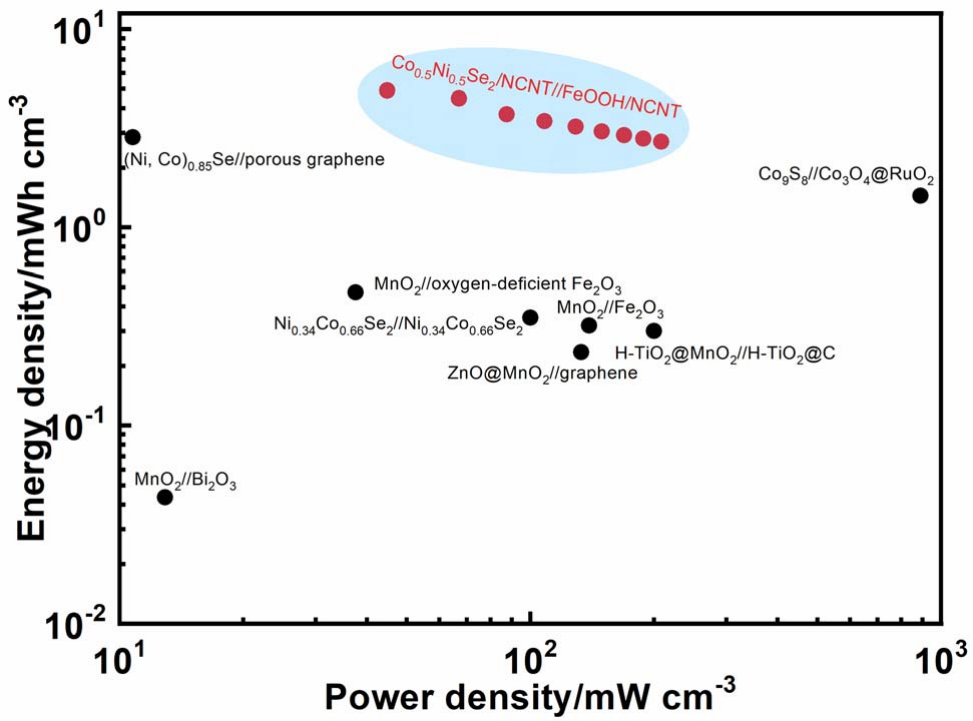
Ragone plot of Co_0.5_Ni_0.5_Se_2_/NCNT//FeOOH/NCNT cell. The performances of the similar energy storage devices reported before were added for comparison.

Ragone plot in Fig. 8 shows energy and power density of Co_0.5_Ni_0.5_Se_2_/NCNT//FeOOH/NCNT cell. It delivers a volumetric energy density of 4.9 mW h cm^−3^ (0.4 mW h cm^−2^) at 44.8 mW cm^−3^ (3.6 mW cm^−2^) and still retains at 2.7 mW h cm^−3^ (0.4 mW h cm^−2^) even at a high power density of 208.1 mW cm^−3^ (16.6 mW cm^−2^). The unexpected energy storage performance, particularly at high power density, is attributed to not only the good electrochemical activity of nickel cobalt selenide component but also the high mass loading and fast ion/electron transport behavior contributed by the coaxial array structure. Moreover, the performance of Co_0.5_Ni_0.5_Se_2_/NCNT//FeOOH/NCNT cell surpasses many best-performing energy storage devices reported before, such as, Ni_0.34_Co_0.66_Se_2_//Ni_0.34_Co_0.66_Se_2_ (0.47 mW h cm^−3^),^57^ H-TiO_2_@MnO_2_//H-TiO_2_@C (0.30 mW h cm^−3^),^58^ MnO_2_//oxygen-deficient Fe_2_O_3_ (0.35 mW h cm^−3^),^59^ MnO_2_//Bi_2_O_3_ (43.4 μW h cm^−2^),^60^ MnO_2_//Fe_2_O_3_ (0.32 mW h cm^−3^),^61^ Co_9_S_8_//Co_3_O_4_@RuO_2_ (1.44 mW h cm^−3^),^62^ and ZnO@MnO_2_//graphene (0.234 mW h cm^−3^),^63^ (Ni, Co)_0.85_Se//porous graphene (2.85 mW h cm^−3^),^50^ CuSe@MnOOH//CuSe@FeOOH (2.9 μW h cm^−3^),^52^*etc.* The gravimetric energy density of Co_0.5_Ni_0.5_Se_2_/NCNT//FeOOH/NCNT cell approximately reaches 46.1 W h kg^−1^ at 421.7 W kg^−1^ and 25.5 W h kg^−1^ at 1958.4 W kg^−1^, depending on active component mass, which also stand at the top level among the state-of-the-art nickel cobalt selenides-based cells including Ni_4.5_Co_4.5_-Se/NPCC//Fe_3_C/CF (47.4 W h kg^−1^ at 1.5 kW kg^−1^),^64^ (Ni, Co)Se_2_/NiCo-LDH//porous carbon (39 W h kg^−1^ at 1650 W kg^−1^),^53^ H-NiCoSe_2_//AC (35 W h kg^−1^ at 188 W kg^−1^),^51^*etc.*

## Conclusion

In summary, we have developed a one-step urea pyrolysis approach to synthesize a highly crystalline NCNT array that serves as a potential scaffold for constructing the coaxial array electrodes. The coaxial array electrodes with the characteristics of large surface area, good electric conductivity, and short ion diffusion pathway exhibited the significantly enhanced electrochemical performance compared to that directly loaded on carbon fiber paper. Moreover, nickel cobalt selenide is demonstrated to exhibit better electrochemical activity than the hydroxide counterpart. Combined with the character of the coaxial array structure and exceptional activity of nickel cobalt selenide, the cell composed of Co_0.5_Ni_0.5_Se_2_/NCNT and FeOOH/NCNT electrodes with a voltage window of 1.6 V exports a maximum volumetric energy density of 4.9 mW h cm^−3^ and can be stably operated for 10 000 cycles. This work provides a facile method for preparing three-dimensional coaxial array electrode with an unprecedent electrochemical performance.

## Conflicts of interest

There are no conflicts to declare.

## Supplementary Material

RA-014-D3RA08635F-s001
