# Supplementary material for: Coaxial nickel cobalt selenide/nitrogen-doped carbon nanotube array as a three-dimensional self-supported electrode for electrochemical energy storage
Source: RSC Adv. 2024 Mar 5;14(11):7710–9. doi: 10.1039/d3ra08635f (PMC10912943; doi:10.1039/d3ra08635f)
Supplement: RA-014-D3RA08635F-s001 [file RA-014-D3RA08635F-s001.pdf]

Electronic Supplementary Material (ESI) for RSC Advances.
This journal is © The Royal Society of Chemistry 2024

Electronic Supplementary Information

Coaxial Nickel Cobalt Selenide/Nitrogen-doped Carbon Nanotube Array as a Three-Dimensional Self-Supported Electrode for Electrochemical Energy Storage

Chen Zhang[1,*], Shang Wang,[2] Junwu Xiao[2]

[1]College of Petroleum Equipment and Electrical Engineering, Dongying Vocational Institute, Dongying, PR China

[2]Key Laboratory of Material Chemistry for Energy Conversion and Storage, Ministry of Education, Hubei Key Laboratory of Material Chemistry and Service Failure, Department of Chemistry and Chemical Engineering, Huazhong University of Science and Technology, Wuhan 430074, China

E-mail: dychenzhang@gmail.com

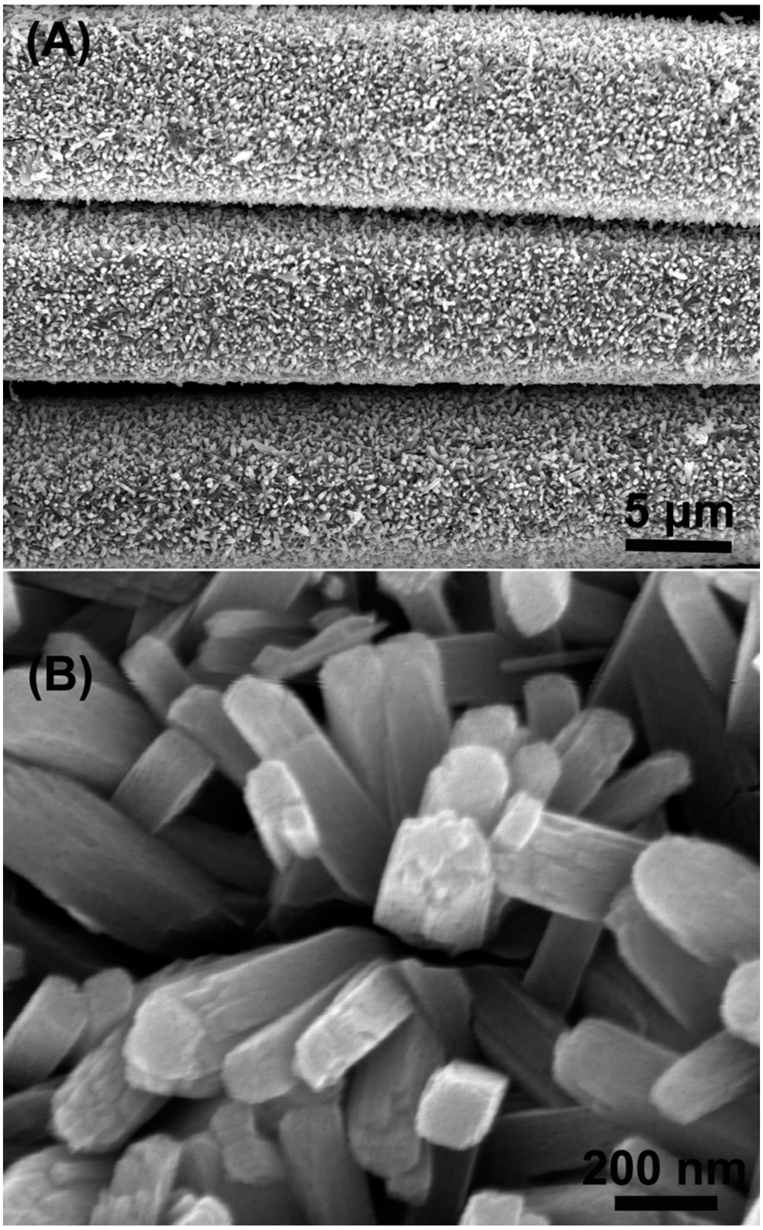

Figure S1. (A) Low and (B) high magnified SEM images of FeOOH nanoarray grown on carbon fiber paper via a hydrolysis of $FeCl_3$.

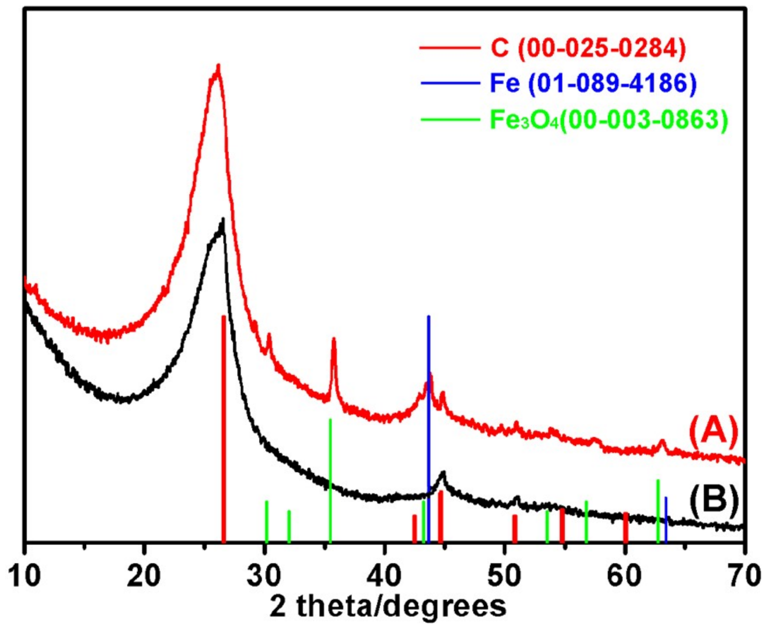

Figure S2. XRD pattern of (A) Fe/Fe_3O_4/NCNT and (B) NCNT array.

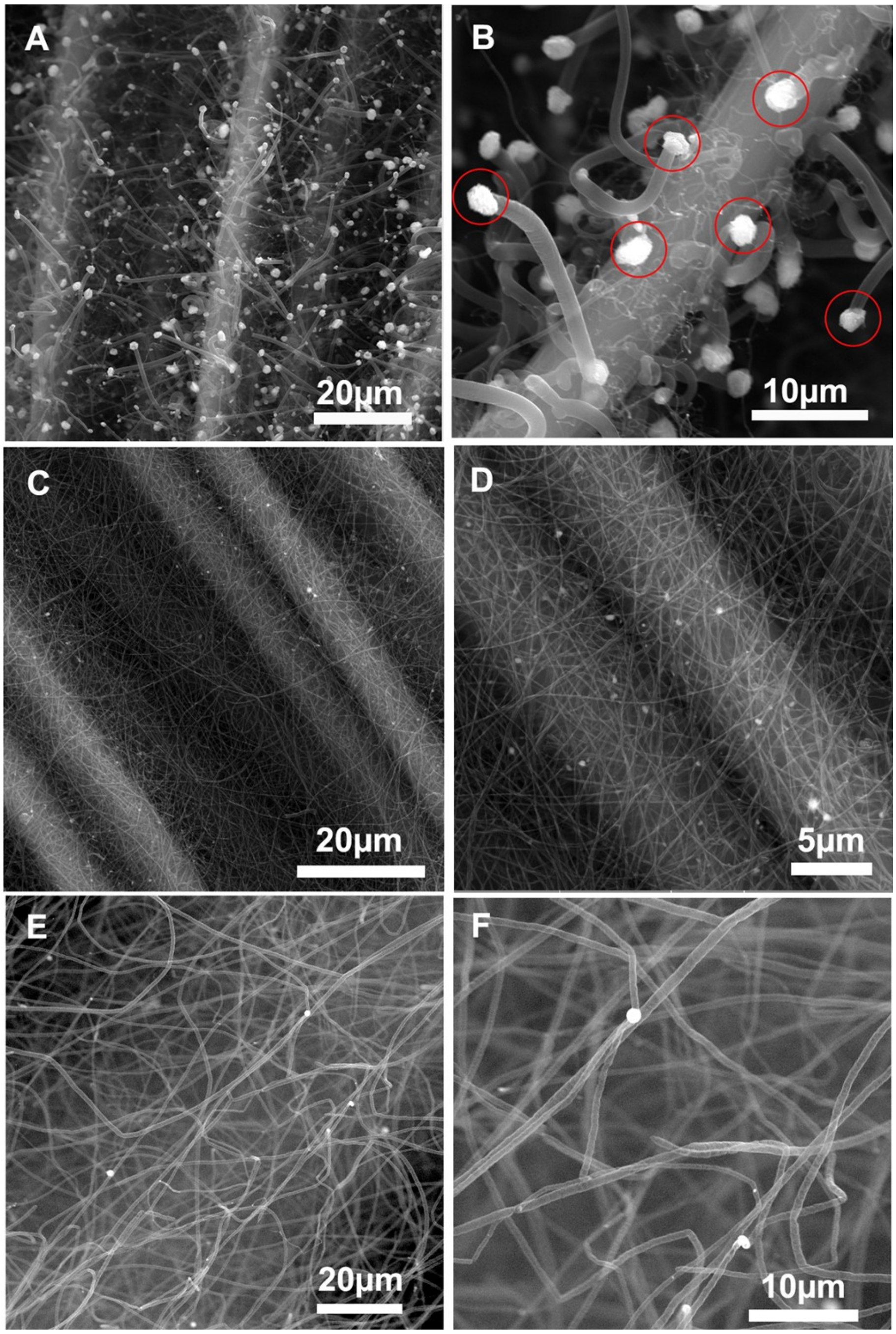

Figure S3. SEM images of Fe/Fe_3O_4/NCNT array formed after different pyrolysis times: (A, B) 10 min, (C, D) 30 min, and (E, F) 60 min.

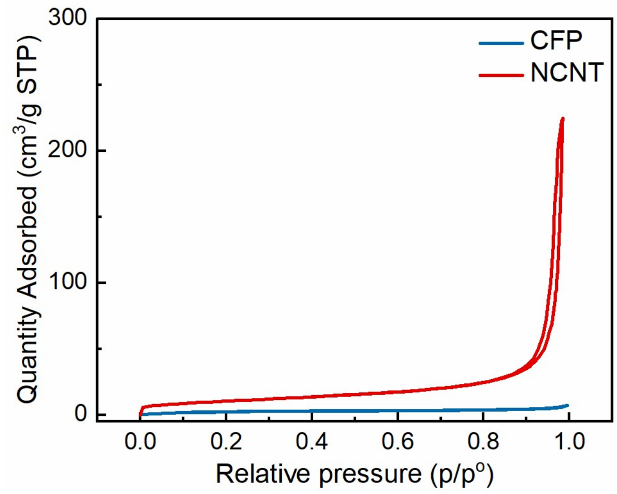

Figure S4. N_2 sorption isotherms of carbon fiber paper (CFP) and NCNT array.

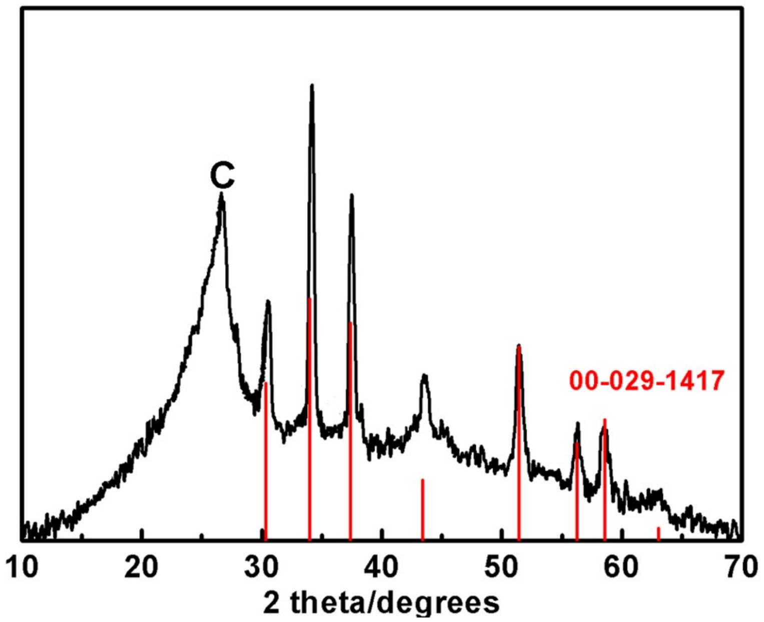

Figure S5. XRD pattern of $Co_{0.5}Ni_{0.5}Se_2$/NCNT, which is in consistent with the standard pattern of $CoSe_2$ (JCPDS 00-029-1417).

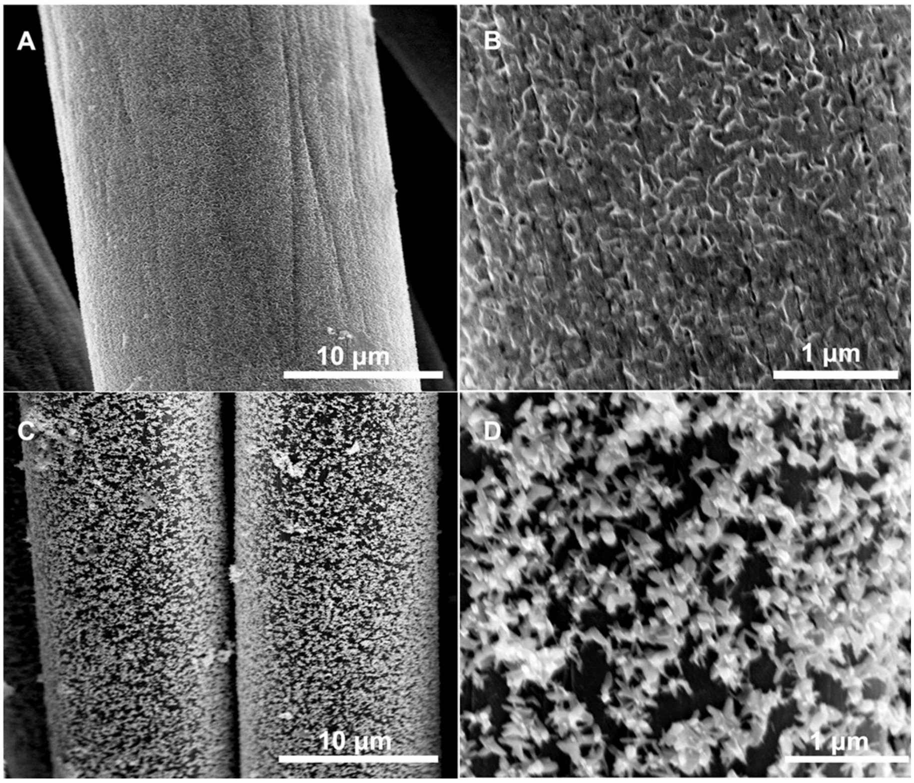

Figure S6. SEM images of (A, B) $Co_{0.5}Ni_{0.5}(OH)_2$/CFP and (C, D) $Co_{0.5}Ni_{0.5}Se_2$/CFP.

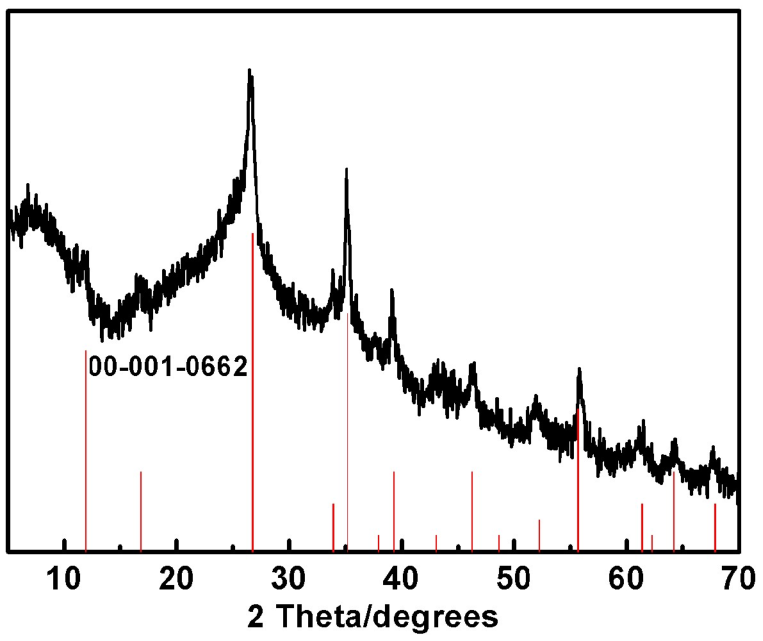

Figure S7. XRD pattern of the coaxial FeOOH/NCNT array, which is in accordance with the standard pattern of FeOOH (JCPDS 00-001-0662).

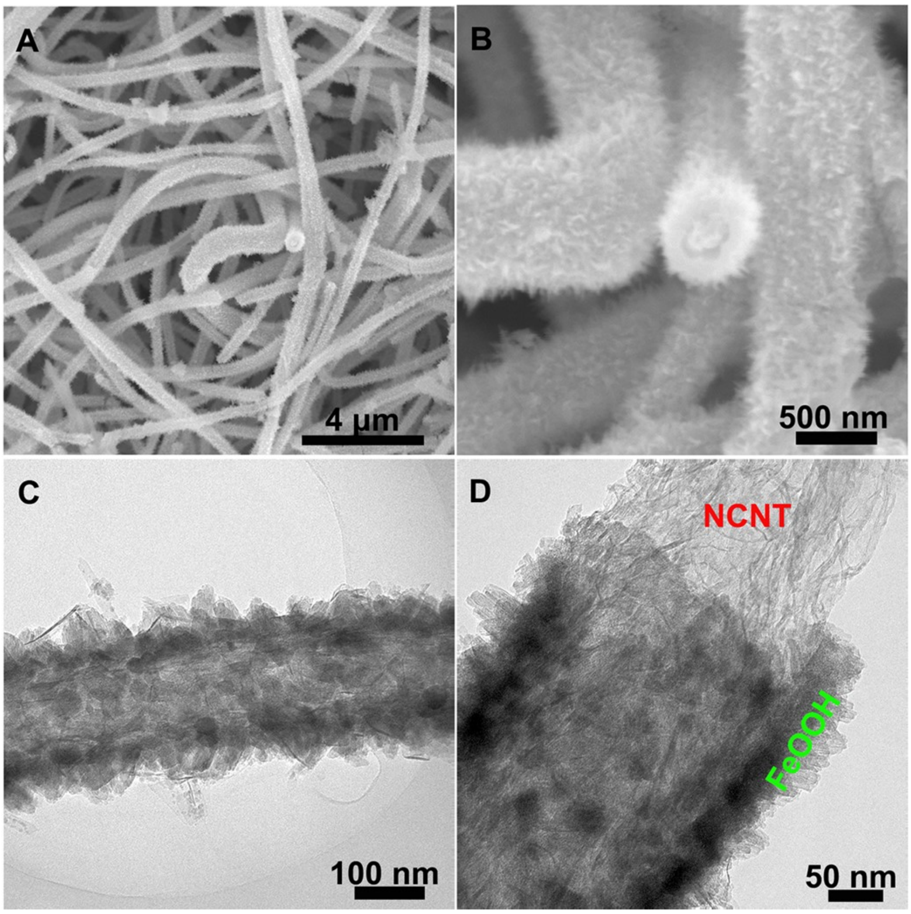

Figure S8. (A, B) SEM and (C, D) TEM images of the coaxial FeOOH/NCNT array.

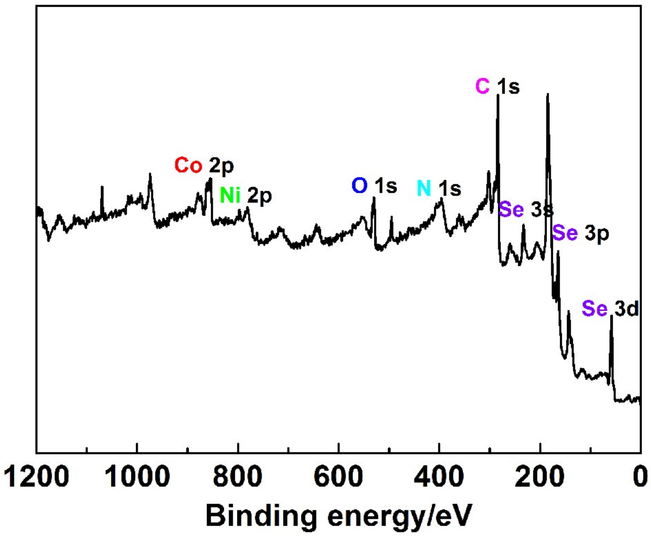

Figure S9. The XPS full spectrum of $Co_{0.5}Ni_{0.5}Se_2$/NCNT.

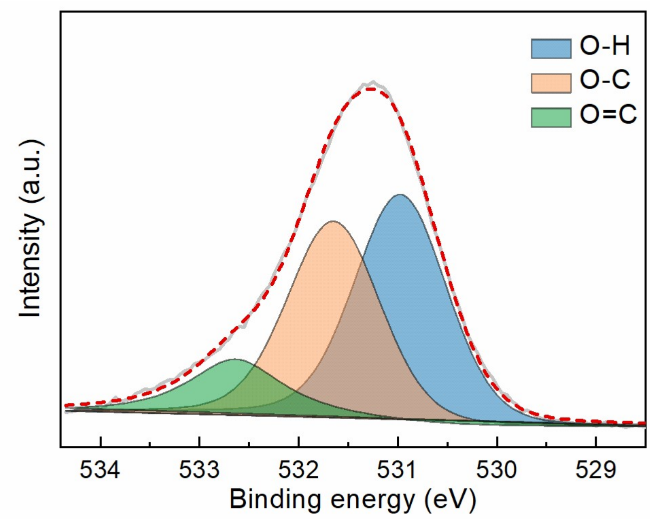

Figure S10. The O 1s XPS fine spectrum for $Co_{0.5}Ni_{0.5}Se_2$/NCNT.

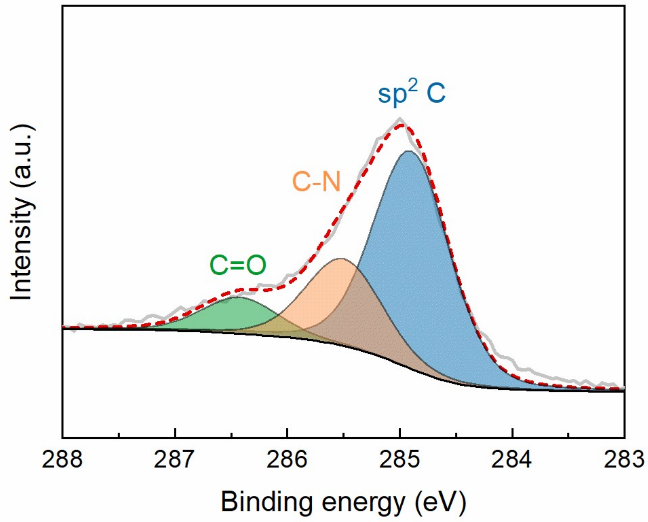

Figure S11. The C 1s XPS fine spectrum for $Co_{0.5}Ni_{0.5}Se_2$/NCNT.

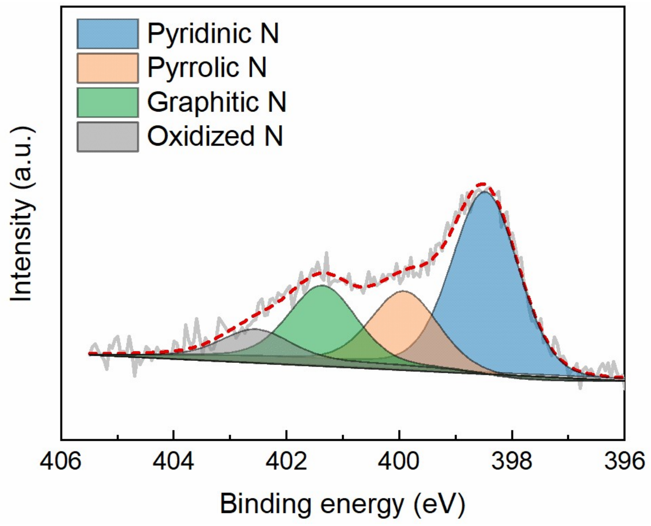

Figure S12. The N 1s XPS fine spectrum for $Co_{0.5}Ni_{0.5}Se_2$/NCNT.

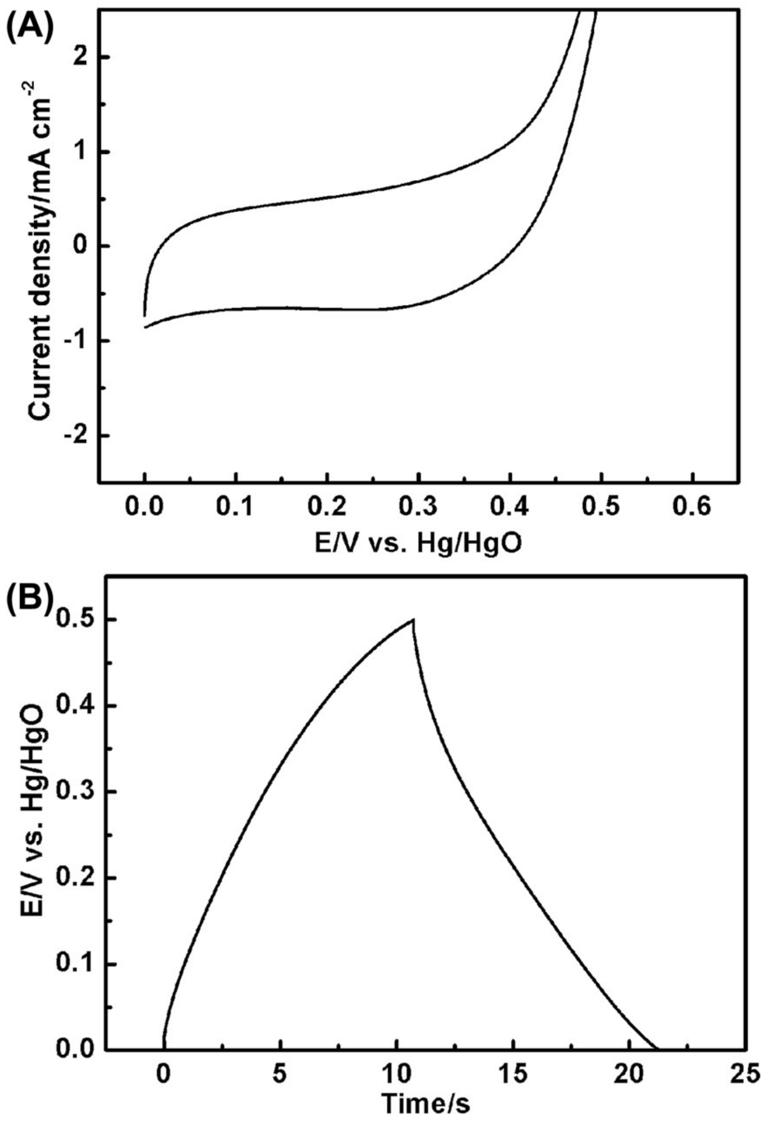

Figure S13. (A) CV curve (10 mV s^{-1}) and (B) galvanostatic charge/discharge curves at 4 mA cm^{-2} of NCNT. The areal capacity is 21.0 mC cm^{-2} at 4 mA cm^{-2}.

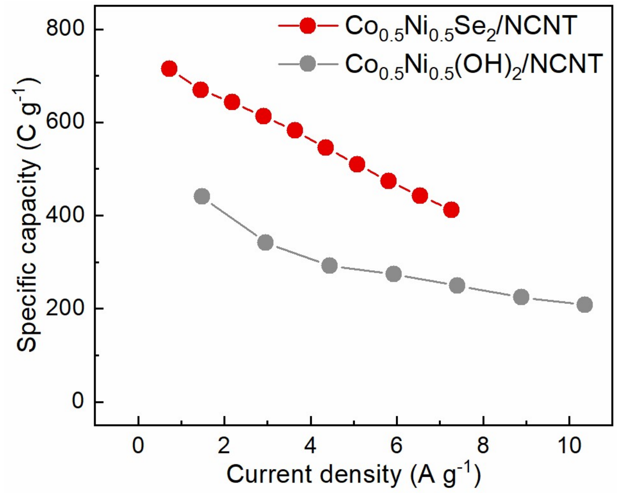

Figure S14. Specific capacity of $Co_{0.5}Ni_{0.5}(OH)_2$/NCNT and $Co_{0.5}Ni_{0.5}Se_2$/NCNT electrodes.

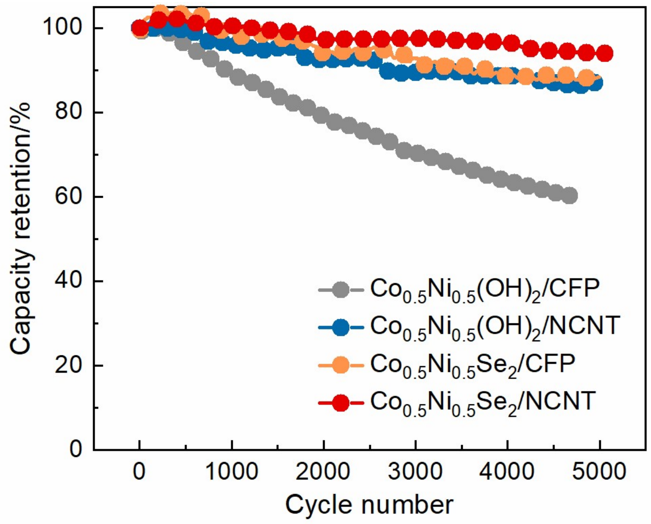

Figure S15. The long-term cycling performances of $Co_{0.5}Ni_{0.5}(OH)_2$/CFP, $Co_{0.5}Ni_{0.5}(OH)_2$/NCNT, $Co_{0.5}Ni_{0.5}Se_2$/CFP, and $Co_{0.5}Ni_{0.5}Se_2$/NCNT at a charging/discharging current of 20 mA cm^{-2}.

Table S1. Comparison of the specific capacity and rate capability of the $Co_{0.5}Ni_{0.5}Se_2$/NCNT coaxial electrode with the Ni-Co-Se electrodes reported before.

Samples	Current density	Specific capacity	Rate capability	Ref.
$Co_{0.5}Ni_{0.5}Se_2$/NCNT	4.0 mA cm^{-2}	3.9 C cm^{-2} (714.5 C g^{-1})	57.6% (4-40)	This work
$Ni_{0.34}Co_{0.66}Se_2$	4.0 mA cm^{-2}	1.31 C cm^{-2}	75% (4-20)	1
Ni-Co-Se	1 A g^{-1}	333.0 C g^{-1}	40.6% (1-20)	2
Ni-Mn-Se	2 A g^{-1}	1221.1 C g^{-1}	78.8% (2-32)	3
$NiCo_{2.1}Se_{3.3}$	1 mA cm^{-2}	371.2 C g^{-1}	63.5% (1-10)	4
$NiCoSe_2$	3 A g^{-1}	375 C g^{-1}	88.0% (3-10)	5
$(Ni_xCo_{1-x})_9Se_8$	5 A g^{-1}	1692.9 C g^{-1}	91.8% (5-30)	6
$NiCoSe_4$	0.5 A g^{-1}	504 C g^{-1}	85.2% (0.5-20)	7
$Ni_{0.5}Co_{0.5}Se_2$	1 A g^{-1}	262 C g^{-1}	56.0% (1-50)	8
$(Ni_{0.33}Co_{0.67})Se_2$	1 A g^{-1}	414.0 C g^{-1}	78.0% (1-30)	9
$NiCoSe_2$	1 A g^{-1}	150.1 C g^{-1}	63.3% (1-20)	10
$NiCoSe_2$	1 A g^{-1}	520 C g^{-1}	53.7% (1-30)	11

References

1. P. Xu, W. Zeng, S. Luo, C. Ling, J. Xiao, A. Zhou, Y. Sun and K. Liao, *Electrochim. Acta*, 2017, **241**, 41-49.
2. Y. L. Liu, C. Yan, G. G. Wang, F. Li, Q. Kang, H. Y. Zhang and J. C. Han, *Nanoscale*, 2020, **12**, 4040-4050.
3. A. Mohammadi Zardkhoshoui, B. Ameri and S. S. Hosseiny Davarani, *Nanoscale*, 2021, **13**, 2931-2945.
4. Y. Wang, W. Zhang, X. Guo, K. Jin, Z. Chen, Y. Liu, L. Yin, L. Li, K. Yin, L. Sun and Y. Zhao, *ACS Appl. Mater. Interfaces*, 2019, **11**, 7946-7953.
5. L. Hou, Y. Shi, C. Wu, Y. Zhang, Y. Ma, X. Sun, J. Sun, X. Zhang and C. Yuan, *Adv. Funct. Mater.*, 2018, **28**, 1705921.
6. P. Yang, Z. Wu, Y. Jiang, Z. Pan, W. Tian, L. Jiang and L. Hu, *Adv. Energy Mater.*, 2018, **8**, 1801392.
7. Z. Xie, D. Qiu, J. Xia, J. Wei, M. Li, F. Wang and R. Yang, *ACS Appl. Mater. Interfaces*, 2021, **13**, 12006-12015.
8. X. Song, C. Huang, Y. Qin, H. Li and H. C. Chen, *J. Mater. Chem. A*, 2018, **6**, 16205-16212.
9. L. Quan, T. Liu, M. Yi, Q. Chen, D. Cai and H. Zhan, *Electrochim. Acta*, 2018, **281**, 109-116.
10. Y. Miao, Y. Sui, D. Zhang, J. Qi, F. Wei, Q. Meng, Y. He, Z. Sun and Y. Ren, *Mater. Lett.*, 2019, **242**, 42-46.
11. L. Wang, Z. Feng, H. Zhang, D. Li and P. Xing, *Nanotechnology*, 2020, **31**, 125403.
